# β-Blocker use is not associated with improved clinical outcomes in women with breast cancer: a meta-analysis

**DOI:** 10.1042/BSR20200721

**Published:** 2020-06-18

**Authors:** Chaoran Li, Tian Li, Runwei Tang, Shuai Yuan, Weihong Zhang

**Affiliations:** Department of Breast Surgery, Baoshan Branch, Shuguang Hospital Affiliated to Shanghai University of Traditional Chinese Medicine, Shanghai 201900, China

**Keywords:** Beta-blocker, Breast cancer, Meta-analysis, Mortality, Recurrence

## Abstract

**Background:** Evidence remains inconsistent regarding the potential influence of β-blocker (BB) use on clinical outcomes in women with breast cancer. We aimed to evaluate the association between BB and prognosis of breast cancer in an updated meta-analysis.

**Methods:** Follow-up studies comparing the clinical outcomes of breast cancer in women with and without use of BB were included by search of PubMed, Embase, and Cochrane’s Library. A random-effect model was used to pool the results.

**Results:** Seventeen observational studies were included. Pooled results did not support a significant association between BB use and breast cancer recurrence (risk ratio [RR] = 0.85, 95% confidence interval [CI]: 0.68–1.07, *P*=0.17), breast cancer related deaths (RR = 0.83, 95% CI: 0.65–1.06, *P*=0.14), or all-cause deaths (RR = 1.01, 95% CI: 0.91–1.11, *P*=0.91) in women with breast cancer. Study characteristics such as sample size, definition of BB use, follow-up durations, adjustment of menopausal status, or quality score did not significantly affect the results. Subgroup analyses showed that BB may be associated with a trend of reduced risk of all-cause deaths in women with breast cancer in prospective studies (two datasets, RR = 0.81, *P*=0.05), but not in retrospective studies (eight datasets, RR = 1.06, *P*=0.16; *P* for subgroup analyses = 0.02).

**Conclusions:** Current evidence from observational studies does not support a significant association between BB use and improved prognosis in women with breast cancer.

## Introduction

Breast cancer is one of the most common malignancies in women [1]. Currently, the incidence of breast cancer remains high, with newly diagnosed cases of about 1.4 million annually [2,3]. Overall, breast cancer is a major global health problem. Although treatments for breast cancer have progressed substantially over the past years, in view of the high morality of the disease, it remains of great clinical significance to develop new medications that confers anticancer efficacy [2,3]. Accumulating experimental data showed that β-adrenoreceptor activation is involved in the pathogenesis and progression of many cancers [4]. Previous studies showed that activation of β-adrenoreceptor axis is associated with up-regulated angiogenesis, activated genes in metastasis and inflammation, and enhanced cell proliferation, thereby mediating tumorigenesis, angiogenesis, and tumor metastasis [5–7]. Accordingly, it has been hypothesized that β-blocker (BB) use may improve the prognosis in cancer patients. Indeed, results of early clinical studies showed that use of BB seemed to be associated with reduced incidence of hepatocellular carcinoma [8] and improved disease-specific survival in patients with prostate cancer [9]. However, studies evaluating the association between BB use and clinical outcomes in women with breast cancer showed inconsistent results [10–26], suggesting that the benefits of BB on cancer survival may be cancer-specific. Some of the previous studies supported that BB use is associated with reduced recurrence or deaths in women with breast cancer [10,11,13,15,17], while others did not [12,14,16,18–26]. Although some meta-analyses were also performed to evaluate the association between BB use and prognosis in women with breast cancer, results of these studies are also inconsistent [27–32]. More importantly, some recently published studies were not included in the previous meta-analyses [24–26]. Therefore, we aimed to systematically evaluate the association between BB use and prognosis in women with breast cancer in an updated meta-analysis. Potential influences of study characteristics on the association were also explored.

## Methods

The MOOSE (Meta-analysis of Observational Studies in Epidemiology) [33] and Cochrane’s Handbook [34] guidelines were followed during the designing, performing, and reporting of the meta-analysis.

### Literature search

Electronic databases of PubMed, Embase, and the Cochrane’s Library were systematically searched using the combination of the following terms: (1) “adrenergic beta antagonist” OR “beta blockers” OR “beta antagonist” OR “beta adrenoreceptor antagonist” OR “beta adrenergic receptor antagonist” OR “beta adrenergic blocking agent” OR “adrenergic beta-1 receptor antagonists” OR “acebutolol” OR “alprenolol” OR “atenolol” OR “betaxolol” OR “bisoprolol” OR “bunolol” OR “bupranolol” OR “Bucindolol” OR “carteolol” OR “celiprolol” OR “Carvedilol” OR “dihydroalprenolol” OR “esmolol” OR “iodocyanopindolol” OR “labetalol” OR “levobunolol” OR “metipranolol” OR “metoprolol” OR “nadolol” OR “Nebivolol” OR “oxprenolol” OR “penbutolol” OR “practolol” OR “pindolol” OR “propranolol” OR “sotalol” OR “timolol”; (2) “breast cancer”; and (3) “survival” OR “prognosis” OR “mortality” OR “death” OR “recurrence” OR ”surgery" OR “operation”. The search was limited to human studies without restriction of the publication language. The reference lists of original and review articles were also analyzed manually. The final literature search was performed on December 24, 2019.

### Study selection

Studies were included if they met the following criteria: (1) published as full-length article; (2) designed as follow-up studies, including randomized controlled trials (RCTs), cohort studies, nested case–control studies, and post-hoc analyses of RCTs, with a minimal follow-up duration of one year; (3) included women with breast cancer; (4) compared the clinical prognosis between breast cancer women of users and non-users of BB; (5) documented the incidence of at least one of the outcomes during follow-up, including breast cancer recurrence, breast cancer related deaths and all-cause deaths; and (6) reported the adjusted risk ratios (RRs, at least adjusted for age) and their corresponding 95% confidence intervals (CIs) for the above outcomes in users and non-users of BB. Reviews, editorials, preclinical studies, cross-sectional studies, and conference abstracts were excluded.

### Data extracting and quality evaluation

Literature search, data extraction, and study quality assessment were independently performed by two authors according to the predefined inclusion criteria. If inconsistencies occurred, discussion with the corresponding author was suggested to resolve these issues. The following data were extracted: (1) name of the first author, publication year, study location, and study design; (2) characteristics and numbers of women with breast cancer, mean ages, definition of BB use, and follow-up period; and (3) number of cases with breast cancer recurrence, breast cancer related deaths, and all-cause deaths during follow-up, and variables adjusted when presenting the RRs. The quality of observational study was evaluated using the Newcastle–Ottawa Scale (NOS) [35]. This scale ranges from 1 to 9 stars and judges the quality of each study regarding three aspects: selection of the study groups; the comparability of the groups; and the ascertainment of the outcome of interest.

### Statistical analyses

The association between BB use and breast cancer recurrence or mortality outcome was measured by RRs in the present study. To stabilize its variance and normalized the distribution, RR data and its corresponding stand error (SE) from each study was logarithmically transformed [34]. The Cochrane’s *Q* test was performed to evaluate the heterogeneity among the include cohort studies [34,36], and the *I*^2^ statistic was also calculated. A significant heterogeneity was considered if *I*^2^ > 50%. A random-effect model was used to pool the results since this model has been indicated to incorporate the potential heterogeneity of the included studies and therefore could provide a more generalized result. Sensitivity analysis by omitting one study at a time was performed to evaluate the stability of the results [34]. Predefined subgroup analyses were performed to evaluate the potential influences of study characteristics on the outcomes, including study design, sample size, definition of BB use, follow-up durations, adjustment of menopausal status, and NOS [37]. Potential publication bias was assessed by visual inspection of the symmetry of the funnel plots, complemented with the Egger regression test [38]. The RevMan (Version 5.1; Cochrane Collaboration, Oxford, U.K.) and STATA software were used for the statistics.

## Results

### Literature search

**Figure 1:**
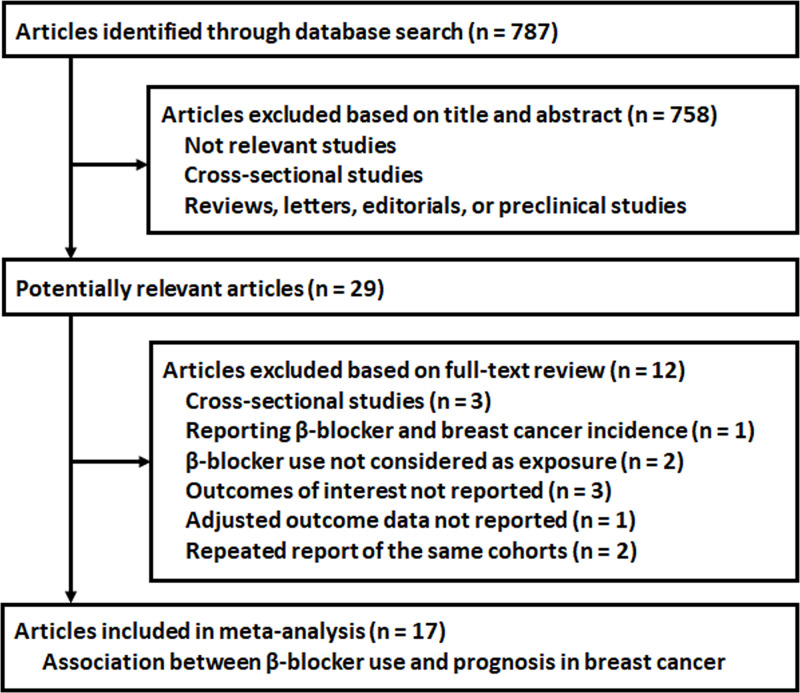
Flowchart of database search and study inclusion

The flowchart of database search was shown in Figure 1. Briefly, 787 studies were obtained from database search, and 758 of them were excluded primarily because they were irrelevance to the aim of the study. For the remaining 29 potential relevant studies that underwent full text review, 12 were further excluded because three of them were cross-sectional, one evaluated the association between BB use and breast cancer incidence, two did not consider BB use as the exposure, three did not report related outcomes, one did not contain adjusted data, and the other two were repeated reports of already included studies. Finally, seventeen studies were included [10–26].

### Study characteristics and quality

Overall, this meta-analysis included 17 studies [10–26] with 75,074 women with breast cancer. Four of them were designed as prospective cohort studies [10,17,18,20], 11 were retrospective cohort studies [11–15,19,21–24,26], and the other two were nested case–control study [16] and post-hoc study [25] respectively. No RCTs were included. Since one study includes two cohorts evaluating the effects of atenolol and propranolol [11], and the other study includes two post-hoc analyses of ROSE/TRIO-012 and BCIRG-005 trials [25], these datasets were included separately. Overall, 19 datasets were available for this meta-analysis. The characteristics of the included cohorts are shown in Table 1. All of these studies were performed in Europe or North America. All of these studies included breast cancer women who received anti-cancer therapy. Women with BB use 1 year prior to the diagnosis or after the diagnosis of breast cancer were considered as BB users. The mean ages of the included women with breast cancer varied between 49 and 76 years. The mean follow-up durations varied from 2.1 to 10.5 years, and outcomes of breast cancer recurrence, breast cancer related deaths, and all-cause deaths were reported. Potential confounding factors, including age, cancer stage at diagnosis, hormonal receptor status, menopausal status, and treatment were adjusted to a varying degree in the included studies. The qualities of the included follow-up studies were generally good, with the NOS ranging from six to eight points.

**Table 1:** Characteristics of the included studies

Study	Country	Design	Patient characteristics	Sample size	Mean age	Definition of BBs use	Follow-up duration	Outcomes reported (*n*)	Outcome validation	Variables adjusted	NOS
					Years		Years				
Powe 2010	U.K.	PC	Women with stage I-II BC	466	57.0	BB use within 1 year prior to the diagnosis of BC	10.0	Recurrence (161) and BC mortality (128)	Medical records	Age, tumor stage, tumor grade, and tumor size	7
Ganz 2011	the US	RC	Women with stage I-IIIA BC	1779	NR	BB use within 1 year prior to the diagnosis of BC	8.2	Recurrence (292), BC mortality (174), and all-cause mortality (323)	Medical records	Age, race, tumor stage, BMI, cancer treatment, HR status, TMX use, and comorbidities of HTN and DM	8
Shah 2011	U.K.	RC	Women with stage I-IV BC	984	NR	BB use within 1 year prior to the diagnosis of BC	4.8	All-cause mortality (NR)	Medical records	Age, smoking status, concurrent medications, and national region	7
Melhem 2011	the US	RC	Women with stage I-III BC	1413	49.4	BB use after the diagnosis of BC	5.1	Recurrence (404) and all-cause mortality (353)	Medical records	Age, race, tumor stage, grade, HR status, BMI, comorbidities, and concurrent medications	8
Barron 2011-pro	Ireland	RC	Women with stage I-IV BC	210	69.0	Use of propranolol within 1 year prior to the diagnosis of BC	3.6	BC mortality (24)	Medical records	Age, tumor stage, tumor grade, and comorbidities	7
Barron 2011-ate	Ireland	RC	Women with stage I-IV BC	1575	71.0	Use of atenolol within 1 year prior to the diagnosis of BC	3.1	BC mortality (414)	Medical records	Age, tumor stage, tumor grade, and comorbidities	7
Botteri 2013	Italy	RC	Postmenopausal women with stage I-III TNBC	800	59.1	BB use prior to the diagnosis of BC	6.0	Recurrence (90) and BC mortality (147)	Medical records	Age, tumor stage, treatment, and concurrent medications	8
Holmes 2013a	the US	PC	Women with stage I-III BC	4661	63.3	BB use after the diagnosis of BC	10.5	BC mortality (292) and all-cause mortality (738)	Medical records	Age, tumor stage, BMI, menopausal status, oral contraceptive use, treatments, and concurrent medications	8
Holmes 2013b	Canada	RC	Women with stage I-IV BC	4019	NR	BB use within 1 year prior to the diagnosis of BC	4.2	All-cause mortality (NR)	Medical records	Age, tumor stage, history of cancer, and area of residence	7
Cardwell 2013	U.K.	NCC	Women with stage I-IV BC	7132	NR	Any BB use after the diagnosis of BC	3.9	BC mortality (1435)	Medical records	Age, tumor stage, TMX, cancer treatments, comorbidities, and concurrent medications	7
Chae 2013	the US	PC	Women with stage I-IV BC	1449	50.3	BB use after the diagnosis of BC	4.6	Recurrence (415), BC mortality (312), and all-cause mortality (359)	Medical records	Age, race, BMI, tumor stage, grade, and concurrent medications	7
Sørensen 2013	Denmark	PC	Women with stage I-III BC	18733	60.2	BB use after the diagnosis of BC	6.8	Recurrence (3414)	Medical records	Age, menopausal status, tumor stage and grade, HR status, cancer treatment, and concurrent medications	8
Boudreau 2014	the US	RC	Women with stage I-II BC	4216	63.0	BB use after the diagnosis of BC	6.3	Recurrence (415)	Medical record	Age, BMI, BC stage, HR status, menopausal status, Charlson comorbidity score, DM, cancer treatments, and concurrent medications	7
Sakellakis 2014	Greece	RC	Women with stage I-III BC	610	60.8	BB use after the diagnosis of BC	3.8	Recurrence (243)	Medical record	Age, tumor stage, and HR status	6
Springate 2015	U.K.	RC	Women with stage I-IV BC	2943	NR	BB use within 1 year prior to the diagnosis of BC	3.2	All-cause mortality (NR)	Medical record	Age, smoking status, concurrent medications, and national region	7
Spera 2017-trio	Canada	Post-hoc	Women with stage IV BC	1144	55.1	BB use after the diagnosis of BC	2.1	All-cause mortality (NR)	Medical record	Age, treatments, and national region	6
Spera 2017-bcirg	Canada	Post-hoc	Women with stage IV BC	3298	NR	BB use after the diagnosis of BC	2.3	All-cause mortality (NR)	Medical record	Age, treatments, and national region	6
Chen 2017	the US	RC	Women with stage I-II BC	14766	NR	BB use after the diagnosis of BC	3.0	Recurrence (627) and BC mortality (237)	Medical record	Age, tumor stage, HR status, cancer treatments, comorbidities, and concurrent medications	7
Musselma 2018	Canada	RC	Women with stage I-IV BC	4876	76.2	BB use within 1 year prior to the diagnosis of BC	4.8	BC mortality (NR) and all-cause mortality (NR)	Medical record	Age, socioeconomic status, and CCI	6

The study by Barron 2011 includes two cohorts evaluating the effects of atenolol and propranolol, and these datasets were included separately.

The study by Spera 2017 includes two post-hoc analyses of ROSE/TRIO-012 and BCIRG-005 trials, and these datasets were included separately.

Abbreviations: BB, β-blockers; BC, breast cancer; BMI, body mass index; CCI, Charlson comorbidity index; DM, diabetes mellitus; HR, hormone receptor; HTN, hypertension; NCC, nested case–control; NOS, the Newcastle–Ottawa Scale; NR, not reported; PC, prospective cohort; RC, retrospective cohort; TMX, tamoxifen; TNBC, triple-negative breast cancer; U.K., United Kingdom; US, United States.

### Association between BB use and recurrence risk of breast cancer

**Figure 2:**
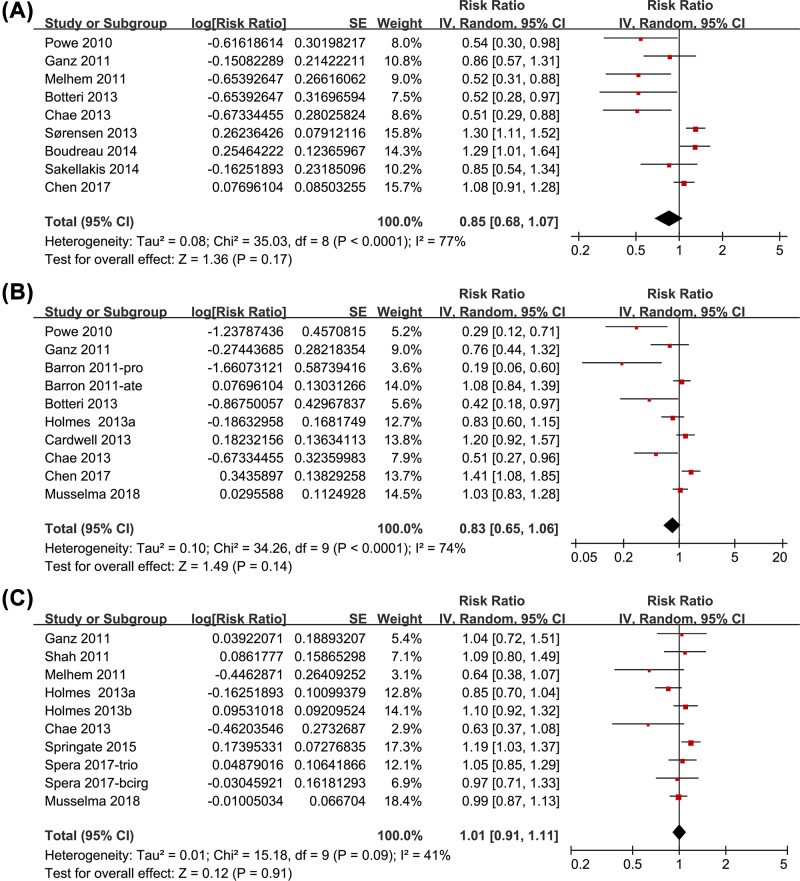
Meta-analysis for the association between BB use and prognosis in women with breast cancer (**A**) Breast cancer recurrence, (**B**) breast cancer related deaths, and (**C**) all-cause deaths

Nine studies [10,12,13,15,17,20–22,24] were included for the meta-analysis of the association between BB use and recurrence risk in women with breast cancer. Significant heterogeneity was detected (*P* for Cochrane’s *Q* test < 0.001, *I*^2^=77%). Pooled results with a random-effect model showed that the association between BB use and breast cancer recurrence was not significant (adjusted RR = 0.85, 95% CI: 0.68–1.07, *P*=0.17; Figure 2A). Results of sensitivity analyses by omitting one study at a time did not significantly change the results (adjusted RR: 0.78–0.91, *P* all > 0.05). Subgroup analysis indicated that the association between BB use and breast cancer recurrence was not significantly affected by study characteristics including study design, sample size, definition of BB use, follow-up durations, adjustment of menopausal status, or NOS (*P* for subgroup difference all > 0.05, Table 2).

**Table 2:** Subgroup analyses

	BC recurrence					BC mortality					All-cause mortality				
Study characteristics	Data number	RR (95% CI)	*I*^2^	*P*_1_	*P*_2_	Data number	RR (95% CI)	*I*^2^	*P*_1_	*P*_2_	Data number	RR (95% CI)	*I*^2^	*P*_1_	*P*_2_
**Study design**															
Prospective	3	0.74 [0.36, 1.52]	88%	0.41		3	0.56 [0.31, 0.99]	65%	0.05		2	0.81 [0.66, 1.00]	5%	0.05	
Retrospective	6	0.88 [0.68, 1.14]	69%	0.34	0.65	7	0.97 [0.76, 1.24]	69%	0.81	0.08	8	1.06 [0.98, 1.14]	10%	0.16	0.02
**Sample size**															
<1000	4	0.77 [0.53, 1.13]	69%	0.11		3	0.55 [0.22, 1.35]	82%	0.19		1	1.09 [0.80, 1.49]	—	0.59	
≥1000	5	0.89 [0.63, 1.25]	82%	0.50	0.58	7	0.90 [0.69, 1.18]	73%	0.46	0.30	9	1.00 [0.90, 1.11]	47%	0.95	0.91
**Definition of BB use**															
Pre-diagnosis	3	0.67 [0.47, 0.94]	20%	0.02		5	0.72 [0.48, 1.08]	76%	0.11		6	1.06 [0.95, 1.18]	32%	0.28	
Post-diagnosis	6	0.96 [0.75, 1.22]	78%	0.73	0.09	5	0.89 [0.62, 1.28]	76%	0.11	0.54	4	0.92 [0.78, 1.08]	28%	0.29	0.14
**Follow-up duration**															
<5 years	3	0.83 [0.55, 1.25]	72%	0.30		6	0.92 [0.68, 1.25]	76%	0.60		6	1.03 [0.93, 1.15]	42%	0.52	
≥5 years	6	0.84 [0.60, 1.17]	81%	0.37	0.96	4	0.63 [0.36, 1.09]	73%	0.10	0.23	4	0.88 [0.68, 1.15]	47%	0.35	0.27
**Adjustment of menopausal status**															
Yes	2	0.87 [0.35, 2.11]	87%	0.75		2	0.66 [0.35, 1.24]	54%	0.20		1	0.85 [0.70, 1.04]	—	0.11	
No	7	0.82 [0.63, 1.07]	73%	0.14	0.91	8	0.87 [0.66, 1.14]	76%	0.31	0.44	9	1.04 [0.94, 1.14]	29%	0.44	0.17
**NOS**															
6	1	0.85 [0.54, 1.34]	—	0.48		1	1.03 [0.83, 1.28]	—	0.79		3	1.00 [0.90, 1.11]	0%	0.88	
7	4	0.87 [0.61, 1.24]	79%	0.45		6	0.79 [0.53, 1.17]	81%	0.24		4	1.09 [0.93, 1.27]	42%	0.16	
8	4	0.78 [0.47, 1.29]	84%	0.33	0.94	3	0.75 [0.56, 1.00]	8%	0.05	0.19	3	0.86 [0.71, 1.04]	12%	0.32	0.17

1. *P* values for subgroup significance;

2. *P* values for subgroup difference;

Abbreviations: BB, β-blocker; BC, breast cancer; CI, confidence interval; HR, hormonal receptors; NOS, the Newcastle–Ottawa Scale; RR, risk ratio.

### Association between BB use and breast cancer related deaths

Meta-analysis with ten datasets from nine studies [10–12,15–18,24,26] showed that the association between BB use and breast cancer related deaths was not significant (adjusted RR = 0.83, 95% CI: 0.65–1.06, *P*=0.14; *I*^2^=74%; Figure 2B). Results of sensitivity analyses by omitting one study at a time did not significantly change the results (adjusted RR: 0.77–0.90, *P* all > 0.05). Subgroup analysis indicated that study characteristics such as study design, sample size, definition of BB use, follow-up durations, adjustment of menopausal status, or NOS did not significantly affect the results (*P* for subgroup difference all > 0.05, Table 2).

### Association between BB use and all-cause deaths

Pooled results of ten datasets from nine studies [12–14,17–19,23,25,26] showed that the association between BB use and all-cause deaths in women with breast cancer was also not significant (adjusted RR = 1.01, 95% CI: 0.91–1.11, *P*=0.91; *I*^2^=41%; Figure 2C). Results of sensitivity analyses by omitting one study at a time did not significantly change the results (adjusted RR: 0.98–1.04, *P* all > 0.05). Subgroup analysis indicated that study design may affect the outcome. Specifically, BB may be associated with a trend of reduced risk of all-cause deaths in women with breast cancer in prospective studies (two datasets, adjusted RR = 0.81, 95% CI: 0.66–1.00, *P*=0.05), but not in retrospective studies (eight datasets, adjusted RR = 1.06, 95% CI: 0.98–1.14, *P*=0.16; *P* for subgroup analyses = 0.02; Table 2). Other study characteristics such as sample size, definition of BB use, follow-up durations, adjustment of menopausal status, or NOS did not significantly affect the results (Table 2).

### Publication bias

**Figure 3:**
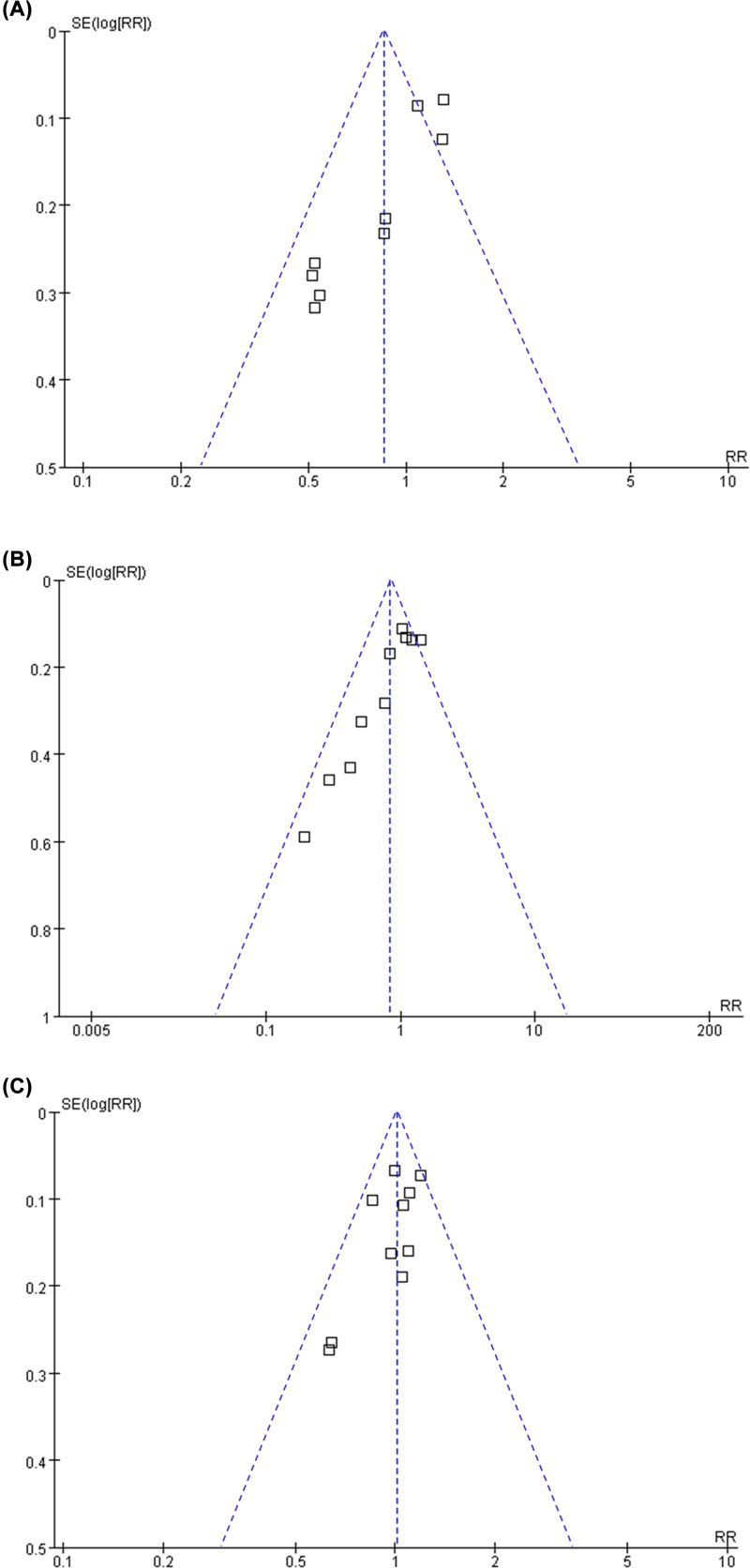
Funnel plots for the meta-analyses of the association between BB use and prognosis in women with breast cancer (**A**) Breast cancer recurrence; (**B**) breast cancer related deaths; and (**C**) all-cause deaths

The funnel plots for the association between BB and risks of recurrence, breast cancer related deaths, all-cause deaths in Figure 3A–C. The plots were symmetrical on visual inspection, suggesting low risks of publication bias. Results of Egger’s regression tests also showed similar results (*P*=0.112, 0.189, and 0.394, respectively).

## Discussion

In the present study, by pooling the results of available observational studies, we found that the BB use is not associated with significantly affected breast cancer recurrence, breast cancer related deaths, or all-cause deaths. Sensitivity analyses by omitting one study at a time did not significantly change the results, demonstrating that the results are stable. Subgroup analyses showed that characteristics such as sample size, definition of BB use, follow-up durations, adjustment of menopausal status, or quality score did not seem to significantly affect the results. Moreover, BB may be associated with a trend of reduced risk of all-cause deaths in women with breast cancer in prospective studies, but not in retrospective studies. Taken together, this updated meta-analysis showed that current evidence from observational studies does not support a significant association between BB use and improved prognosis in women with breast cancer. However, in view of the potential anticancer effects of BB evidenced in experimental studies and the potential limitations of the retrospective studies, qualified RCTs are needed to eventually determine if BB has additional clinical benefits in women with breast cancer.

Previous meta-analyses showed inconsistent results regarding the association between BB use and prognosis in women with breast cancer. An early meta-analysis with seven studies published before 2015 showed that BB was associated with a significantly reduced risk of breast cancer related deaths, but not for cancer recurrence or total mortality [27]. However, a subsequent pooled analysis with individual data from eight European cohorts published before 2016 showed that use of propranolol or non-selective BB was not associated with improved survival in women with breast cancer [39]. In addition, a subsequent meta-analysis in 2016 with four cohort studies showed that post-diagnosis BB use was associated with significantly improved overall survival in women with breast cancer, but not for pre-diagnosis BB use [29]. On the contrary, a later meta-analysis performed in 2017 with six studies showed that BB use was not beneficial for breast cancer recurrence, cancer specific mortality, or overall deaths [31]. These previous meta-analyses generally include four to eight studies and the results of the meta-analyses were unstable. Moreover, due to the limited datasets available, the authors were unable to evaluate the influences of study characteristics on the outcome by performing comprehensive subgroup analyses. Our current meta-analysis has the following strengths in this regard. First, our study included 19 datasets from 17 studies with 75,074 women with breast cancer. The overall sample size of the meta-analysis is significant larger than the previous studies. Moreover, nine to ten datasets were available for each outcome and the stability of the results were validated by sensitivity analyses. Third, only studies with adjusted data regarding the association between BB use and clinical outcomes in women with breast cancer were included to reduce the potential influence of confounding factors. Finally, subgroup analyses were performed for each study, and the results showed that study characteristics such as sample size, definition of BB use, follow-up durations, adjustment of menopausal status, or quality score did not seem to significantly affect the results. Taken together, this updated-analysis showed that BB use is unlikely to be associated with significantly affected clinical prognosis in women with breast cancer.

In contrast with the non-significant association between BB use and breast cancer prognosis as evidenced by the observational studies, experimental studies and small-scale RCTs consistently showed potential beneficial effects of BB in breast cancer. A recent study showed that Carvedilol treatment could suppress malignant proliferation of mammary epithelial cells *in vitro* through inhibition of the reactive oxygen species-mediated phosphoinositide 3–kinase/protein kinase B pathway [40]. Moreover, β2-adrenergic signaling was shown to promote cell migration in cultured breast cancer cell lines by up-regulating expression of the metastasis-associated molecule Ly6/PLAUR domain-containing protein 3 [41]. Interestingly, propranolol treatment of breast cancer cells is associated with disrupted cell cycle progression, steady state levels of cyclin, increased p53 levels, and enhanced cellular apoptosis [42]. These findings strongly support an anticancer efficacy of BB in breast cancer by inhibition cancer cell proliferation and migration, as well as inducing apoptosis. More importantly, two phase-II RCTs including early stage breast cancer patients showed that compared to placebo, perioperative treatment with propranolol significantly inhibited multiple cellular and molecular pathways related to metastasis and disease recurrence in excised tumors and sequential blood samples [43,44]. These findings support the need for larger phase III clinical trials powered to detect the impact of β-blockade on breast cancer recurrence and survival.

Our study has limitations that should be noticed when interpreting the results. First, although data of adjusted RR were combined, residual factors may also exist that may confound the association between BB and prognosis of breast cancer, such as concurrent use of other medications that may affect the prognosis of breast cancer, such as metformin [45] and statins [46]. Second, all of the included studies were performed in Europe or North America. It has been suggested that the survival of women with breast cancer may vary by ethnicity [47]. Therefore, the association between BB use and prognosis in breast cancer should be performed in women with other race and ethnicity. Third, we are unable to evaluate whether the selective or the non-selective BB may influence the prognosis of women with breast cancer differently since data regarding these categories of medications were rarely reported. Future studies are warranted to determine the potential difference between the influences of the selective and the non-selective BB use on the prognosis of breast cancer. Fourth, since breast cancer is a hormonal-dependent cancer, and the change of hormonal status during menopausal may affect the characteristics of the tumor, it is reasonable to evaluate the influence of hormonal status and the menopausal status on the association between BB use and prognosis in breast cancer. However, due to the limited information available from the included studies, we were unable to determine whether the association between BB use and prognosis in breast cancer differs according to hormone status of the cancer, menopausal status of the patients, or individual medications of BB. Further studies are warranted. Finally, most of the included studies were of retrospective design, which may expose to recall bias. Qualified RCTs are needed to eventually determine if BB has additional clinical benefits in women with breast cancer.

In conclusion, our meta-analysis showed that current evidence from observational studies did not support a significant association between BB use and improved prognosis in women with breast cancer. Considering the limitations of observational studies, qualified RCTs are needed to eventually determine if BB has additional clinical benefits in women with breast cancer.

## Abbreviations

BB: β-blockers
BMI: body mass index
CCI: Charlson comorbidity index
DM: diabetes mellitus
HR: hormone receptor
HTN: hypertension
NCC: nested case–control
NOS: the Newcastle–Ottawa Scale
TMX: tamoxifen
TNBC: triple-negative breast cancer
